# Clinical and genetic correlates of islet-autoimmune signatures in juvenile-onset type 1 diabetes

**DOI:** 10.1007/s00125-019-05032-3

**Published:** 2019-11-21

**Authors:** Laura A. Claessens, Joris Wesselius, Menno van Lummel, Sandra Laban, Flip Mulder, Dick Mul, Tanja Nikolic, Henk-Jan Aanstoot, Bobby P. C. Koeleman, Bart O. Roep

**Affiliations:** 1 Department of Immunohaematology and Blood Transfusion, Leiden University Medical Center, Leiden, the Netherlands; 2 Department of Medical Genetics, University Medical Center Utrecht, Utrecht, the Netherlands; 3 Diabeter, Center for Pediatric and Adolescent Diabetes Care and Research, Rotterdam, the Netherlands; 4 Department of Diabetes Immunology, Diabetes & Metabolism Research Institute, Beckman Research Institute, National Medical Center, City of Hope, 1500 E Duarte Road, Duarte, CA 91010 USA

**Keywords:** Autoimmune disease, Autoreactive T cells, Disease endotypes, Disease heterogeneity, Epitope spreading, Immunotherapy, Islet autoantigen, Patient heterogeneity, Personalised medicine, Precision medicine

## Abstract

**Aims/hypothesis:**

Heterogeneity in individuals with type 1 diabetes has become more generally appreciated, but has not yet been extensively and systematically characterised. Here, we aimed to characterise type 1 diabetes heterogeneity by creating immunological, genetic and clinical profiles for individuals with juvenile-onset type 1 diabetes in a cross-sectional study.

**Methods:**

Participants were HLA-genotyped to determine *HLA-DR-DQ* risk, and SNP-genotyped to generate a non-HLA genetic risk score (GRS) based on 93 type 1 diabetes-associated SNP variants outside the MHC region. Islet autoimmunity was assessed as T cell proliferation upon stimulation with the beta cell antigens GAD65, islet antigen-2 (IA-2), preproinsulin (PPI) and defective ribosomal product of the insulin gene (INS-DRIP). Clinical parameters were collected retrospectively.

**Results:**

Of 80 individuals, 67 had proliferation responses to one or more islet antigens, with vast differences in the extent of proliferation. Based on the multitude and amplitude of the proliferation responses, individuals were clustered into non-, intermediate and high responders. High responders could not be characterised entirely by enrichment for the highest risk HLA-*DR3-DQ2/DR4-DQ8* genotype. However, high responders did have a significantly higher non-HLA GRS. Clinically, high T cell responses to beta cell antigens did not reflect in worsened glycaemic control, increased complications, development of associated autoimmunity or younger age at disease onset. The number of beta cell antigens that an individual responded to increased with disease duration, pointing to chronic islet autoimmunity and epitope spreading.

**Conclusions/interpretation:**

Collectively, these data provide new insights into type 1 diabetes disease heterogeneity and highlight the importance of stratifying patients on the basis of their genetic and autoimmune signatures for immunotherapy and personalised disease management.

**Electronic supplementary material:**

The online version of this article (10.1007/s00125-019-05032-3) contains peer-reviewed but unedited supplementary material, which is available to authorised users.

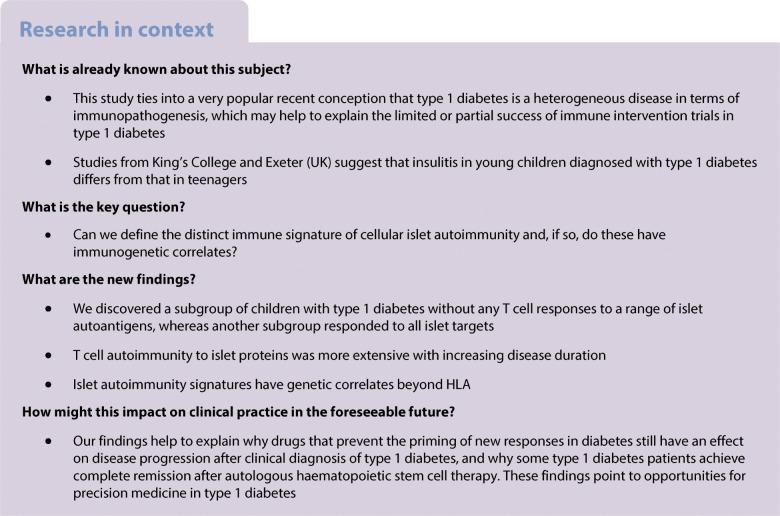



## Introduction

Type 1 diabetes is an autoimmune disease characterised by a loss of functional insulin-producing beta cells in the pancreas. Recently, heterogeneity of the disease has become more appreciated, but it has not yet been extensively and systematically characterised. Individuals differ in their disease pathogenesis, disease progression, genetic background and response to immune intervention therapy [1]. Clinically, patients show large variations in age at disease onset, glycaemic control, C-peptide production, exogenous insulin use, and time of onset and severity of complications. This implies that there is a need for patient stratification and precision medicine.

Both genetic and environmental factors contribute to a loss of immune tolerance towards beta cell antigens, such as glutamate decarboxylase 65 (GAD65), islet antigen-2 (IA-2) and preproinsulin (PPI). The HLA region at 6p21 accounts for approximately 50% of disease susceptibility, which is in part conferred by HLA class I, but mostly by HLA class II [2]. HLA-DRB1*04-DQA1*0301-DQB1*0302 (*DR4-DQ8*) and *HLA*-DRB1*0301-DQA1*0501-DQB1*0201 (*DR3-DQ2*) haplotypes predispose to disease development [3, 4]. Individuals that have both haplotypes (*DR3-DQ2/DR4-DQ8*) are at the highest risk [5]. In contrast, several haplotypes show evidence for protection from disease. In particular HLA-DRB1*1501-DQA1*0102-DQB1*0602 is believed to cause dominant protection. There are currently also over 50 non-HLA genomic regions that show moderate, yet significant association with the disease, with odds ratios (ORs) ranging from 1.02 to 3.28 [6–10]. Genetic risk scores (GRSs) are now becoming widely used for individual disease-risk prediction for common genetic diseases [11]. For type 1 diabetes, a GRS combines genetic risk of HLA- and non-HLA-associated variants in an individual quantitative score that can serve as the best disease prediction. Such a GRS was successful in discerning type 1 diabetes from monogenetic and type 2 diabetes, and predicting type 1 diabetes risk [12–16]. Besides its relation to risk for disease, genetic risk quantified by the type 1 diabetes GRS may also contribute to predicting progression towards disease, as well as to immunological and clinical heterogeneity after disease onset.

A better understanding of disease heterogeneity is pivotal for improving clinical research and personalised disease management. Therefore, the aim of this study was to characterise disease heterogeneity in a cross-sectional cohort of individuals with juvenile-onset type 1 diabetes by creating immunological, clinical and genetic profiles.

## Methods

### Blood donors

Peripheral blood was collected cross-sectionally from 80 consenting individuals with type 1 diabetes who consecutively reported for their regular medical check-up at the Diabeter Clinic in Rotterdam (the Netherlands), without any inclusion/exclusion criteria (participants demographics are listed in Table 1). Peripheral blood mononuclear cells (PBMC) were isolated and subsequently tested for the presence of autoreactive T cells using our standard T cell proliferation assay (see below). HbA_1c_ measurements were recorded at and around (± 12 months) the date of blood sampling, and presence of IA-2 and GAD autoantibodies was analysed at disease diagnosis. Complications and the development of associated autoimmunity up until the date of blood sampling were included in our analyses, such as celiac disease, microalbuminuria, hyperthyroidism, hypertension and kidney failure. All participants signed informed consent and the study was approved by the Medical Ethics Committee of Diabeter, Rotterdam and the Leiden University Medical Center.

**Table 1:** Demographics

Demographic	Value	Range
Sex (male/female)	44/36	–
Mean age at blood sampling, years	17.9 ± 6.6	4.6–41.8
Mean age at disease onset, years	9.2 ± 4.8	0.7–23.4
Mean disease duration, years	8.6 ± 7.1	0.0–32.0

Data are reported as *n* or mean ± SD

### HLA genotyping, SNP genotyping and GRS computation

DNA was isolated from frozen granulocytes or leftover PBMCs with the DNeasy Blood & Tissue Kit (Qiagen Benelux, Venlo, the Netherlands). DNA concentration was determined by NanoDrop (Thermo Fisher Scientific, Waltham, MA, USA) and samples were concentrated to 50 ng/μl. HLA class I and II loci (*HLA-A*, *-B*, *-C*, *-DRB1*, *-DQA1*, *-DQB1*) were genotyped at four-digit resolution. SNP genotyping was performed on the Infinium ImmunoArray-24 v2 BeadChip Kit (Illumina, Eindhoven, the Netherlands). To test the cumulative effect of non-HLA type 1 diabetes-associated SNP variants, we computed a GRS in individuals, based on previous studies [12–14]. GRS is the sum of the number of risk alleles (0, 1 or 2) multiplied by the natural logarithm of the OR for each SNP variant, divided by the total number of SNP variants, where ‘i’ is the index number of the SNPs used to construct the GRS:

$$ {\mathrm{GRS}}_{\mathrm{non}-\mathrm{HLA}}=\sum \limits_{\mathrm{i}=1}^{{\mathrm{SNP}}_{\mathrm{total}}}\frac{\left({\mathsf{\log}}_{\mathit{\mathsf{e}}}\left({\mathrm{OR}}_{\mathrm{SNP}\mathrm{i}}\right)\times {\mathrm{copy}}_{\mathrm{SNP}\mathrm{i}}\right)}{{\mathrm{SNP}}_{\mathrm{total}}} $$

Chromosome X SNP variants in male individuals were counted as 0 or 2, which assumes a dominant risk effect in the hemizygous state. Ninety-three non-HLA risk-SNP variants were included in the score (electronic supplementary materials [ESM] Table 1). rs12720356 typing failed in three individuals. ORs were obtained from www.immunobase.org (accessed January 2017).

### T cell proliferation assay

A T cell proliferation assay was performed on freshly isolated PBMC to investigate autoimmunity towards the beta cell-derived antigens GAD65, PPI, IA-2, and the recently discovered defective ribosomal product of the insulin gene (INS-DRIP) [17]. Since INS-DRIP is a beta cell-specific neoantigen that is produced increasingly during stress, T cell responses to this antigen could potentially reflect beta cell stress. The human recombinant proteins GAD65, PPI, IA-2 and INS-DRIP were produced as previously described [17, 18]. PBMC were seeded (150,000/well) in round-bottomed 96-well microculture plates (Greiner, Nürtingen, Germany) and cultured for 5 days in Iscove’s Modified Dulbecco’s Media (IMDM) containing 10% (vol./vol.) human serum, at 37°C in 5% (vol./vol.) CO_2_ in a humidified atmosphere. Cells were cultured in triplicates in medium alone, with 10 μg/ml recombinant GAD65, PPI, IA-2 or INS-DRIP, or with recombinant IL-2 (35 U/ml; Genzyme, Cambridge, MA, USA) as a positive control. In the final 16 h of culture, 50 μl RPMI 1640 (Dutch modification; Gibco; Thermo Fisher Scientific) containing 18,500 Bq ^3^H-thymidine (DuPont NEN, Boston, MA, USA) was added per well. After the cells were harvested on filters with an automated harvester, proliferation was determined by measurement of ^3^H-thymidine incorporation in an automatic liquid scintillation counter (LKB Instruments, Gaithersburg, MD, USA). All results are calculated as mean counts per min (CPM) in the presence of antigen (CPM_antigen_) and compared with medium alone (CPM_medium_). Stimulation index (SI) = mean CPM_antigen_/mean CPM_medium_. SI ≥ 3 was considered a positive response.

### Data analysis

Hierarchical clustering, principal component analysis (PCA) and heat maps of T cell proliferation data were computed in R Version 3.3.3 (Auckland, New Zealand). SI values were natural-log transformed prior to analysis. Clustering was computed using Euclidean distance and complete linkage methods with the functions ‘dist()’ and ‘hclust()’. Clustering was visualised with ‘fviz_dend()’ to create a dendrogram and ‘fviz_cluster()’ to create a PCA plot, both from the package ‘factoextra’. Heat maps were generated with ‘Heatmap’ from the package ‘ComplexHeatmap’. The 3D-plot with regression plane was generated in R with ‘scatter3D’ from the package ‘plot3D’ and ‘lm’. Spearman correlation analysis of all parameters was performed and visualised in R with ‘rcorr()’ from the package ‘Hmisc’ and ‘corrplot()’ from the package ‘corrplot’. Missing values were deleted pairwise. PCA of all parameters was performed with ‘PCA()’ and visualised with ‘fviz_pca_biplot’ from the package ‘factoextra’. Missing values were deleted listwise. GraphPad Prism 7 (La Jolla, CA, USA) was used to create all other figures and perform corresponding statistical analyses. In univariate analyses, individuals with missing data were excluded individually. For non-parametric data, medians were shown and compared between groups with Dunn’s multiple comparison test. For parametric data, means were shown and compared between groups with Tukey’s multiple comparison test. Proportions of individuals were compared between groups using a χ^2^ test. Adobe Illustrator (San Jose, CA, USA) was used for final processing of all figures.

## Results

### Multitude and amplitude of cellular islet autoimmunity identifies distinct participant clusters

**Fig. 1:**
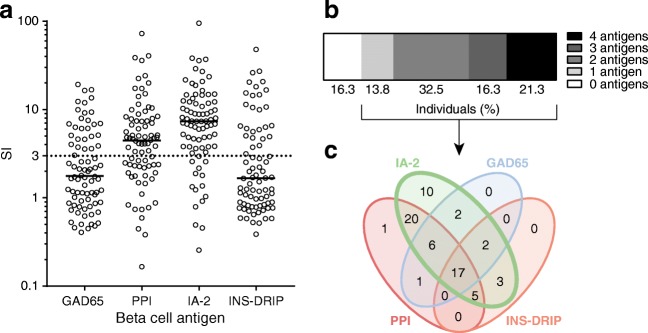
Multitude and amplitude of beta cell-specific T cell proliferation. (**a**) SI values for proliferation of PBMC upon stimulation with the beta cell antigens GAD65, PPI, IA-2 and INS-DRIP in *n* = 80 individuals. Horizontal bars indicate group medians. SI ≥ 3 is considered positive and is indicated by the dotted line. The *y*-axis is on a log_10_ scale. (**b**) Percentage of individuals with a positive proliferation response against *n* = 0, *n* = 1, *n* = 2, *n* = 3 or *n* = 4 beta cell antigens. (**c**) Venn diagram of the number of individuals with positive proliferation responses against single beta cell antigens or all possible combinations of antigens. Red, PPI; green, IA-2; blue, GAD65; orange, INS-DRIP

To assess immunological heterogeneity, T cell proliferation against beta cell antigens, GAD65, IA-2, PPI and INS-DRIP, was measured. For all beta cell antigens, a wide range of proliferation was noted (Fig. 1a). High T cell proliferation was observed for IA-2- and PPI-stimulated PBMC (median SI, 7.4 and 4.5, respectively). Of all participants, 81.3% and 62.5% had positive (SI ≥ 3) IA-2- and PPI-specific T cell proliferative responses, respectively, whilst 35.0% and 33.8% of individuals had positive GAD65- and INS-DRIP-specific responses, respectively. Considering the total number of positive T cell responses against beta cell antigens within a single individual, most individuals responded to at least one antigen (83.8%), where 70.1% responded to two or more beta cell antigens (Fig. 1b). Of these, all responded to IA-2 alone or in combination with other beta cell antigens, with the exception of one individual responding to PPI only and another individual responding to both PPI and GAD65 (Fig. 1c).

**Fig. 2:**
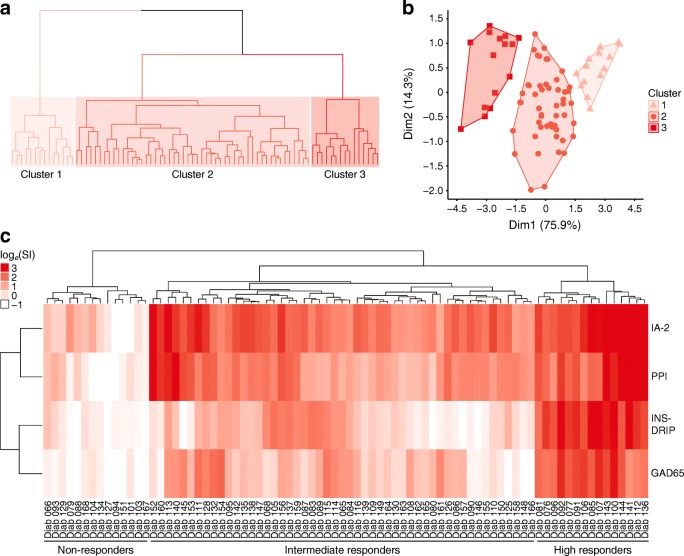
Hierarchical clustering of individuals based on beta cell-specific T cell proliferation. Stimulation indices of beta cell-specific T cell proliferation were log_*e*_-transformed [log_*e*_(SI)] and clustered with Euclidian distance and complete linkage methods. Clustering was visualised in a dendrogram (**a**), PCA plot (**b**) and heat map (**c**). In (**a**), the height of the dendrogram is relative to the dissimilarity (the higher the dendrogram, the more dissimilar the data). The different clusters are highlighted by different shades of red. (**b**) PCA plot of principal component 1 (Dim1) and principal component 2 (Dim2). Colours for clusters 1, 2 and 3 in (**b**) correspond to those in (**a**). In (**a**) and (**b**): cluster 1, non-responders; cluster 2, intermediate responders; cluster 3, high responders. (**c**) Heat map with participant ID

Combining the amplitude and multitude of beta cell-specific T cell proliferation in a hierarchical clustering method and PCA (Fig. 2) identified three distinct participant clusters. One distinct cluster of participants did not display T cell proliferation against the tested beta cell antigens (‘non-responders’; *n* = 14). A second distinct cluster of patients had high T cell proliferative responses against all four beta cell antigens (‘high responders’; *n* = 15). The majority of patients showed mixed responses, with varying T cell proliferation against 1–3 beta cell antigens (‘intermediate responders’; *n* = 51). Age at blood sampling and sex did not differ significantly between these clusters (data not shown).

### High responders can, in part, be characterised by high *HLA-DR-DQ* and non-HLA genetic risk

**Fig. 3:**
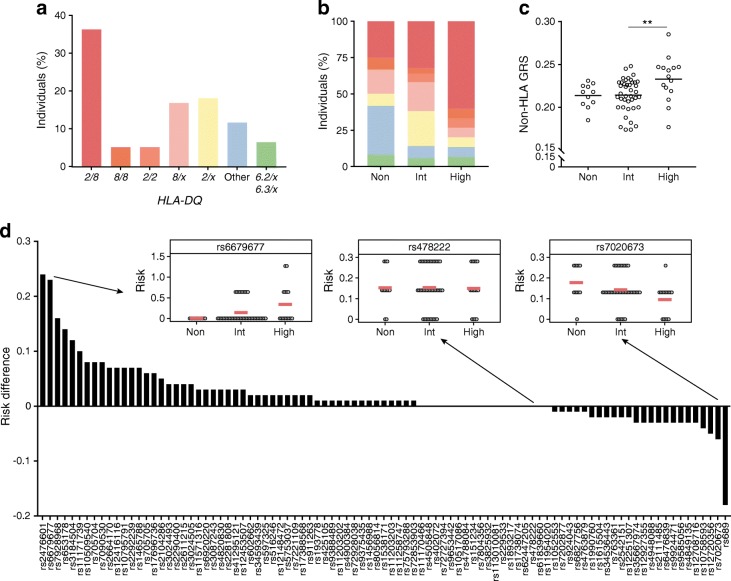
HLA and non-HLA genetic profile of different types of immune responders. (**a**) Distribution of *HLA-DR-DQ* haplotypes in 77 individuals, from high- to low-risk: *DR3-DQ2/DR4-DQ8* (*2/8*); *DR4-DQ8/DR4-DQ8* (*8/8*); *DR3-DQ2/DR3-DQ2* (*2/2*); *DR4-DQ8/x* (*8/x*); *DR3-DQ2/x* (*2/x*); *DR15-DQ6.2/x* (*6.2/x*); *DR13-DQ6.3/x* (*6.3/x*); or other. (**b**) Percentage of individuals per *HLA-DR-DQ* haplotype in the different responder groups. Significance was tested using χ^2^ test. Colours in (**b**) correspond with the haplotypes shown in (**a**). (**c**) Non-HLA GRS based on 93 type 1 diabetes-associated SNP variants for 67 individuals and plotted per responder group. Horizontal bars indicate group means. **p < 0.01, Tukey’s multiple comparison test. (**d**) SNP variants ranked from high to low based on the difference between mean genetic risk in high responders and mean genetic risk in non- and intermediate responders combined for each SNP variant. Inset graphs are representative examples of SNP variants, showing genetic risk among responder groups; horizontal red bars indicate group means. High, high responders; Int, intermediate responders; Non, non-responders

*HLA-DR-DQ* is an important risk factor for developing type 1 diabetes and may also play a role in causing the observed immunological heterogeneity that accompanies this disease. Individuals were classified as high or low risk based on the following genotypes (listed from high to low risk): *DR3-DQ2/DR4-DQ8*, *DR4-DQ8/DR4-DQ8*, *DR3-DQ2/DR3-DQ2*, *DR4-DQ8/x*, *DR3-DQ2/x*, other (non-associated *DR-DQ*) and *DR15-DQ6.2/x* or *DR13-DQ6.3/x*, where *x* is a non-associated *DR-DQ* (non-*DR3-DQ2*, non-*DR4-DQ8*, non-*DR15-DQ6.2* and non-*DR13-DQ6.3*). Of all individuals, 81.8% had a genotype that confers increased risk for developing this disease, with most individuals having the highest risk *DR3-DQ2/DR4-DQ8* genotype (36.4%; Fig. 3a).

No significant differences were observed in the proportion of individuals per *HLA-DR-DQ* genotype between types of responders (*p* = 0.098; Fig. 3b). The high responder group did appear to have more *DR3-DQ2/DR4-DQ8* genotypes than non- or intermediate responders (60.0% vs 25.0% and 32.0%, respectively), though this difference was not significant (*p* = 0.096). Conversely, a small proportion of high and intermediate responders had a neutral or protective *HLA-DR-DQ* genotype (13.3% and 14.0%, respectively) compared with 41.7% of non-responders. However, this difference did not reach significance (*p* = 0.072).

To assess non-HLA genetic risk, we generated a GRS including 93 non-HLA type 1 diabetes-SNP variants (non-HLA GRS) for each individual (ESM Table 1). High responders had a higher non-HLA GRS than intermediate and non-responders (*p* = 0.009 and *p* = 0.053, respectively; Fig. 3c). Non- and intermediate responders were not distinguishable based on the mean non-HLA GRS.

### Non-HLA SNP ranking identifies SNP variants that are overrepresented in high responders

It is conceivable that not all 93 SNP variants contributed positively to distinguishing high responders from non- and intermediate responders. To estimate which SNP variants were relevant, we calculated the genetic risk per SNP variant per individual using the following equation:

$$ {\mathrm{Individual}}_{\mathrm{x}}\hbox{--} {\mathrm{SNP}}_{\mathsf{i}}={\mathsf{\log}}_{\mathit{\mathsf{e}}}\left({\mathrm{OR}}_{\mathrm{SNP}\mathrm{i}}\right)\times {\mathrm{copy}}_{\mathrm{SNP}\mathrm{i}} $$

where, Individual_x_ refers to an individual participant, SNP_i_ to an individual SNP, log_*e*_(OR_SNPi_) to the natural logarithm of the OR of that SNP, and copy_SNPi_ to the number of risk alleles the individual has for that SNP.

Then, we calculated the mean genetic risk per SNP variant for the non-, intermediate and high responders. SNP variants showed three different risk patterns in responders: (1) equal risk in all responders; (2) increasing risk with increasing response; and (3) decreasing risk with increasing response (ESM Fig. 1). SNP variants were ranked by importance by calculating, per SNP variant, the difference between mean genetic risk in high responders and the mean genetic risk in non- and intermediate responders combined (risk difference SNP_i_ = mean SNP_i high responders_ – mean SNP_i non+intermediate responders_) (Fig. 3d). SNP variants with a high, positive risk difference included rs2476601 (*PTPN22*), rs6679677 (*PTPN22*), rs7928968 (*INS*), rs653178 (*SH2B3*), rs3184504 (*SH2B3*), rs11171739 (*ERBB3*), rs10509540 (*RNLS*), rs705704 (*ERBB3*) and rs7090530 (*IL2RA*).

### T cell proliferation to beta cell autoantigens and clinical presentation

**Fig. 4:**
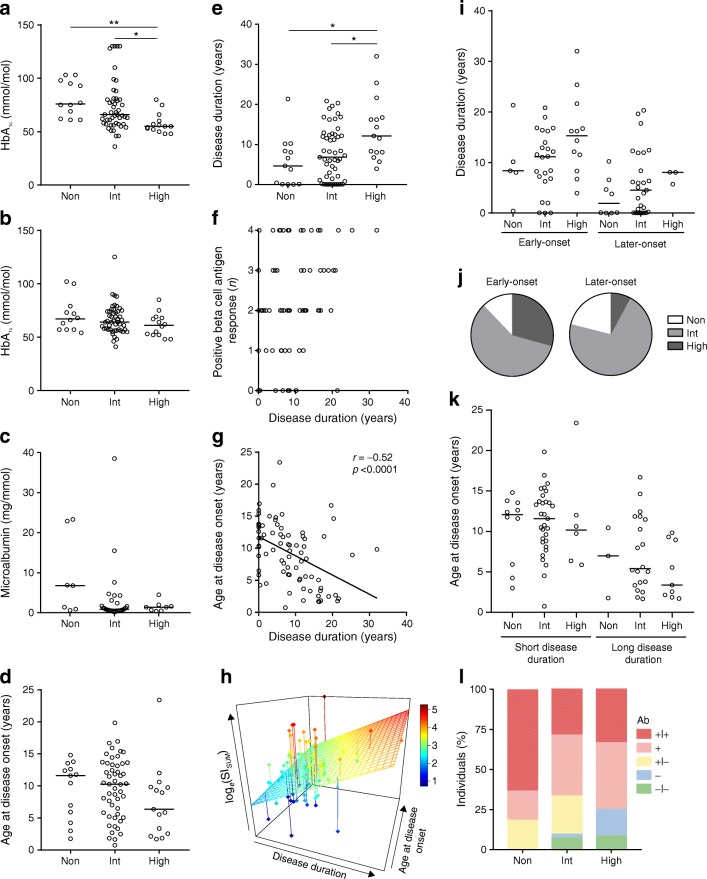
Clinical profile of different types of immune responders. (**a**) HbA_1c_ at the date of blood sampling in 75 individuals. (**b**) Mean HbA_1c_ of measurements taken up to 12 months before or after the date of blood sampling. (**c**) Microalbumin at date of blood sampling in 44 individuals. (**d**) Age at disease onset per responder group. (**e**) Disease duration at the date of blood sampling per responder group. (**f**) Disease duration plotted against the number of positive beta cell-specific T cell responses per individual. The number of beta cell antigens a single individual responded to increased with longer disease duration (*p* = 0.035). (**g**) Scatter plot of disease duration and age at disease onset. Spearman correlation was performed and the linear regression line was plotted. (**h**) 3D plot of log_*e*_(SI_SUM_), disease duration and age at disease onset. The regression plane was plotted. The colour bar represents log_*e*_(SI_SUM_) values. (**i**) Disease duration per responder group for individuals stratified into early-onset (<10 years; *n* = 41) and later-onset (≥10 years; *n* = 38) disease. (**j**) Pie-chart of proportion of responders in the early-onset (<10 years) and later-onset (≥10 years) groups. (**k**) Age at disease onset per responder group for individuals stratified into short disease duration (<10 years; *n* = 47) and long disease duration (≥10 years; *n* = 32). (**l**) Percentage of individuals (*n* = 68) that were double-positive for IA-2 and GAD autoantibodies (+|+), single-positive when only one was measured (+), single-positive when both are measured (+|−), single-negative when only one was measured (−) and double-negative (−|−). For (**a**–**e**) and (**i**) and (**k**): horizontal bars indicate group medians; significance was tested using Dunn’s multiple comparison test. For (**j**) and (**l**), significance was tested using a χ^2^ test. *p < 0.05, **p < 0.01. Ab, antibodies; High, high responders; Int, intermediate responders; Non, non-responders

To determine whether the islet-autoimmune signatures correlated with clinical parameters, HbA_1c_ measurements at the date of blood sampling were collected. If there was no HbA_1c_ measurement at the exact date of blood sampling, the nearest measurement was taken. Intriguingly, HbA_1c_ was significantly lower in high responders compared with intermediate and non-responders (*p* = 0.011 and *p* = 0.001, respectively; Fig. 4a). HbA_1c_ showed an inverse correlation with age at blood sampling (*p* = 0.005; ESM Fig. 2a). To confirm age was not a confounder, individuals were stratified (<18 years old and ≥18 years old). In both groups, high responders had a lower HbA_1c_ than non-responders (<18 years, *p* = 0.039; ≥18 years, *p* = 0.046; ESM Fig. 2b). In young individuals, high responders also had lower HbA_1c_ than intermediate responders (*p* = 0.049). To get a sense of chronic glycaemic control, the mean of all HbA_1c_ measurements up to 12 months before and after the date of blood sampling were calculated. There were no significant differences in mean HbA_1c_ between responders (Fig. 4b). Additionally, the occurrence of complications and associated autoimmunity was evaluated. Microalbumin measured at the date of blood sampling varied from 0.3 to 38.5 mg/mmol creatinine and did not differ significantly between responder groups (Fig. 4c). One individual, a non-responder, was treated once for hypertension and another individual, an intermediate responder, was treated once for hyperthyroidism. Two individuals were also diagnosed with celiac disease (a non-responder and an intermediate responder).

### Longer disease duration is associated with increasing beta cell-specific T cell proliferation

Age at disease onset did not differ significantly between responders (Fig. 4d). Disease duration increased with increasing beta cell-specific T cell proliferation; high responders had significantly longer disease duration than intermediate and non-responders (*p* = 0.047 and *p* = 0.014, respectively; Fig. 4e). Likewise, the number of beta cell antigens a single individual responded to increased with longer disease duration (*p* = 0.035; Fig. 4f). However, disease duration and age at disease onset were inversely correlated (*r* = −0.52, *p* < 0.0001; Fig. 4g). To assess whether high responders had a longer disease duration independent of their age at disease onset, a 3D-plot with regression plane was generated of disease duration, age at disease onset and beta cell-specific T cell proliferation (Fig. 4h). For this purpose, the latter was converted into a continuous variable by taking the sum of all individual proliferation responses (SI_SUM_ = SI_GAD65_ + SI_PPI_ + SI_IA-2_ + SI_INS-DRIP_) and its natural logarithm (log_*e*_(SI_SUM_)). We observed a positive change for disease duration and log_*e*_(SI_SUM_) for all ages at disease onset. Correspondingly, stratification of individuals into a group of early-onset (<10 years) and later-onset (≥10 years) disease pointed to similar increases in disease duration with increasing beta cell autoimmunity in both groups, though this was no longer significant (Fig. 4i). However, the proportion of non-, intermediate and high responders in each group was different (*p* = 0.046; Fig. 4j). Likewise, to assess whether islet autoimmunity and age at disease onset were influenced by disease duration, individuals were stratified into short (<10 years) and long (≥10 years) disease duration. There were no significant differences in age at disease onset between responders in both strata (Fig. 4k). An important clinical parameter that is used in the prediction and diagnosis of type 1 diabetes is the presence of islet autoantibodies. IA-2 and/or GAD autoantibodies were measured at diagnosis. Presence of these antibodies at diagnosis did not correlate significantly with beta cell-specific T cell proliferation at the time of blood sampling (Fig. 4l), namely 63.6% of non-responders were double autoantibody-positive compared with 28.6% of intermediate responders and 33.3% of high responders (*p* = 0.095; Fig. 4l).

### Multi-parameter analyses demonstrate vast complexity and heterogeneity

**Fig. 5:**
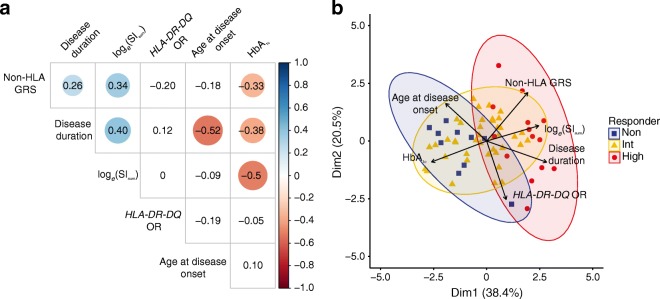
Multi-parameter analyses. (**a**) Spearman correlation of all variables (*n* = 80 individuals). Indicated values represent Spearman correlation coefficient (*r*). Values are encircled if correlation is significant (*p* < 0.05). The size and colour of circles represent strength of correlation and significance (larger circles indicate greater significance; blue indicates positive correlations, whilst red indicates negative correlations). (**b**) PCA plot of principal component 1 (Dim1) and principal component 2 (Dim2) using log_*e*_(SI_SUM_), *HLA-DR-DQ* OR, non-HLA GRS, age at disease onset, disease duration and HbA_1c_ at date of sampling (*n* = 64 individuals)

Correlation analysis of all included parameters revealed further co-linearity. *HLA-DR-DQ* genotypes were converted to their corresponding ORs to generate a continuous variable for use in the correlation analysis (ESM Table 2). The majority of ORs for the *HLA-DR-DQ* genotypes were known from literature [3]. If there was no reported OR, an estimation was made based on the risk of the *HLA-DR-DQ* haplotypes. Microalbumin and islet autoantibodies were excluded from the analysis. Supporting our univariate cluster analysis above, log_*e*_(SI_SUM_) showed significant correlations with disease duration, non-HLA GRS and HbA_1c_, but not with age at disease onset and *HLA-DR-DQ* (Fig. 5a). Moreover, non-HLA GRS was significantly correlated with disease duration and HbA_1c_, of which the latter were also correlated to one another.

Clustering individuals based on T cell proliferation against beta cell-specific antigens allowed the identification of three distinct clusters. However, after adding genetic and clinical parameters, individuals no longer clustered separately by PCA (Fig. 5b). Superimposing the previously identified clusters showed that there was a small overlap of non- and high responders. Intermediate responders no longer appeared separate, but overlapped completely with either non- or high responders, or both.

## Discussion

Type 1 diabetes disease heterogeneity has become more generally appreciated, but has not yet been extensively and systematically characterised, nor has this been implemented to any considerable extent through stratification into immune intervention therapy other than age at disease onset. In this study, we show widespread heterogeneity at the level of cellular islet autoimmunity. We found vast differences in the amplitude and multitude of beta cell-specific T cell proliferation and identified different types of responders. It is generally believed that most immune-mediated beta cell damage has happened before clinical onset of the disease and that the rate of active autoimmunity declines thereafter, in line with islet-autoantibody titres [19]. However, we show the opposite, with almost all individuals responding to at least one of the tested beta cell antigens. This is in line with the observation that most individuals still have beta cells years after type 1 diabetes onset that may be targeted by the immune system when the disease is progressing [20, 21]. Our data suggest a key role for the beta cell antigen IA-2 in disease pathogenesis, with virtually all individuals responding to IA-2 alone or in combination with other tested beta cell antigens. Interestingly, one in six individuals did not respond to any beta cell antigen. It is possible that other beta cell antigens are involved here, or that beta cell-specific T cells are anergic or residing in the insulitic lesion or pancreas-draining lymph nodes.

Non- and high responders were less frequent, while the majority of individuals showed highly mixed responses. These islet-autoimmune signatures could, in part, be defined by genetic parameters, but high responders had an only slightly larger proportion of individuals with the highest risk *HLA-DR3-DQ2/DR4-DQ8* than non- and intermediate responders. It is important to consider that almost all individuals with type 1 diabetes carry *HLA-DR-DQ*-associated risk, which affects the probability of defining differences in *HLA-DR-DQ* risk among them. However, non-HLA genetic risk was a strong determinant for islet autoimmunity, with high responders having a significantly higher non-HLA GRS. Interestingly, not all SNP variants in our non-HLA GRS predisposed individuals to increased islet autoimmunity, while all associated with risk for type 1 diabetes. SNP variants that showed the strongest positive change with increased islet autoimmunity in our ranking method were linked to *PTPN22*, *INS*, *SH2B3*, *ERBB3*, *RNLS* and *IL2RA*. Variants in these genes were previously associated with the development of autoantibodies, disease development and progression from presenting with autoantibodies to type 1 diabetes onset in at-risk individuals [12, 22–26]. However, how these SNPs exactly contribute to increased T cell autoimmunity is unclear. The susceptible allele of rs2476601 (*PTPN22*) decreases T cell and B cell receptor signalling, potentially impairing the establishment of immune tolerance [27]. *SH2B3* and *IL2RA* also play a role in regulating immune-cell signalling and proliferation and could, thereby, contribute to an imbalance in (autoreactive) T cell responsiveness [28]. Conversely, SNPs in *INS* and *ERBB3* are thought to affect insulin expression and beta cell function [28]. *ERBB3* is also expressed in antigen-presenting cells and has been associated with T cell stimulatory capacity [29]. Thus, SNPs in these genes might impact T cell responsiveness by affecting autoantigen release/presentation and central tolerance, as well as indirectly influencing immune-cell function.

Clinically, increased islet autoimmunity did not reflect in worse blood glucose control, as measured by HbA_1c_. Indeed, increased islet autoimmunity was associated with lower HbA_1c_ at time of blood sampling. However, individuals with similar HbA_1c_ levels can have large differences in mean glucose, questioning its predictive value [30]. Only five individuals experienced a type 1 diabetes-related complication or developed associated autoimmunity, as expected for young individuals with short disease duration. Additional clinical parameters, such as insulin requirements and C-peptide, may provide more insight into possible clinical consequences or correlates of increased islet autoimmunity.

We found a positive relation between disease duration and beta cell antigen response. Age at disease onset did not associate significantly with islet autoimmunity in both the entire cohort and after adjustment for disease duration. This could be due to limited participant number and/or the nature of our cross-sectional study in which disease duration and age at disease onset are, per definition, inversely correlated. This raises the question as to whether increased islet autoimmunity led to an earlier age at disease onset and, consequently, longer disease duration in individuals with similar age or, alternatively, whether a longer disease duration allowed for development of progressive islet autoimmunity. Additionally, the disease is believed to progress differently in individuals who are diagnosed during childhood vs individuals who are diagnosed during adolescence [31]. Stratifying individuals by early- and later-onset suggested that, in both groups, a longer disease duration was associated with increased islet autoimmunity, an indication of ongoing priming of immune cells and epitope spreading to multiple beta cell antigens long after clinical manifestation of disease. This perhaps unexpected observation supports the notion that autoimmunity may be sustained by both functional and non-functional beta cells and is in line with the clinical efficacy of drugs that prevent the priming of immune responses (e.g., abatacept), even if offered after diagnosis [32].

The presence of islet autoantibodies increases the risk of developing type 1 diabetes [33]. However, the predictive role of islet autoantibodies at diagnosis for the clinical course of the disease remains elusive. Here, we did not find a correlation between autoantibody prevalence at diagnosis and beta cell-specific T cell proliferation later in life. If anything, autoantibody positivity at diagnosis was slightly more common in T cell non-responders, though this was not significant. Having varying time periods between autoantibody measurements and T cell analyses, we were unable to address the relationship between simultaneously existing autoantibody and T cell responses. However, an inverse correlation between humoral and cellular islet autoimmunity has been described before [34, 35].

An additional potential confounding limitation of our cross-sectional study design (in contrast to longitudinal measures) is that we cannot make any predictions about disease progression with regard to the immunological and genetic profiles observed, nor can we distinguish between age at onset and disease duration. It would be crucial to determine stability or changes in islet-autoimmune signatures over time, such as whether immune signatures of given participants are stable or transient. Additionally, the individuals included in our study were at different stages of disease. Longitudinal studies of individuals with new-onset type 1 diabetes, with multiple follow-up periods to test both autoimmunity and beta cell function are required to determine immunological and genetic heterogeneity at the time of disease onset and their relationship with disease progression. Furthermore, even though relatively large in size, our cohort of 80 individuals was not sufficient to fully encompass the immunological heterogeneity of type 1 diabetes. Validation in new cohorts and larger numbers of participants in heterogeneity studies will allow for even more robust multivariate statistical analyses to reveal interactions between variables and their contribution in creating meaningful patient signatures.

With this study, we attempted to capture type 1 diabetes heterogeneity at different ages and disease duration, prompting further research and definition of disease endotypes. Type 1 diabetes is a complex, multifactorial disease and, with each additional risk factor and measure for analysing individuals with this disease taken into account, additional heterogeneity will likely be found. Improving patient stratification is pivotal for gaining a better understanding of disease heterogeneity, identification of different disease mechanisms and, ultimately, for the assignment of personalised disease management.

## Electronic supplementary material

ESM (PDF 1556 kb)

## Data Availability

All data are available from the authors on request.

## Abbreviations

CPM: Counts per min
GRS: Genetic risk score
IA-2: Islet antigen-2
INS-DRIP: Defective ribosomal product of the insulin gene
PBMC: Peripheral blood mononuclear cells
PCA: Principal component analysis
PPI: Preproinsulin
SI: Stimulation index
