# Supplementary material for: Clinical and genetic correlates of islet-autoimmune signatures in juvenile-onset type 1 diabetes
Source: Diabetologia. 2019 Nov 21;63(2):351–61. doi: 10.1007/s00125-019-05032-3 (PMC6946733; doi:10.1007/s00125-019-05032-3)
Supplement: Supplementary file 1 — (PDF 1556 kb) [file 125_2019_5032_MOESM1_ESM.pdf]

ESM Table 1 – Type 1 diabetes-associated SNP variants in non-HLA GRS

Region	Position	Genes	rsID	Risk allele	OR (risk allele)
T1D 1p13.2	chr1:113288123-114009223	PTPN22 PHTF1	rs2476601	G>A	1.93
		PTPN22 PHTF1	rs6679677	C>A	1.89
T1D 1q32.1	chr1:206709013-206867593	IL10	rs3024493	A>C	1.22
		IL10	rs3024505	A>G	1.18
T1D 2q24.2	chr2:162104363-162504293	IFIH1	rs2111485	A>G	1.18
		IFIH1	rs1990760	G>A	1.15
		IFIH1	rs35667974	G>A	1.69
T1D 2q33.2	chr2:203749263-203951852	CTLA4	rs3087243	A>G	1.18
		CTLA4	rs11571316	A>G	1.22
T1D 2q13	chr2:110225473-111045908		rs4849135	A>C	1.12
T1D 2p23.3	chr2:24795892-25264789	EFR3B	rs478222	T>A	1.15
T1D 2q11.2	chr2:99926678-100432653	AFF3	rs9653442	A>G	1.11
T1D 3p21.31	chr3:46061249-46593299	CCR5	rs113010081	G>A	1.18
T1D 4q32.3	chr4:165571673-165682849		rs2611215	G>A	1.18
T1D 4q27	chr4:122017479-122644147	IL21 IL2 ADAD1	rs75793288	C>G	1.15
		IL21 IL2 ADAD1	rs6827756	G>A	1.13
		IL21 IL2 ADAD1	rs4505848	A>G	1.12
		IL21 IL2 ADAD1	rs17388568	G>A	1.19
T1D 4p15.2	chr4:26026175-26130530		rs10517086	G>A	1.09
T1D 5p13.2	chr5:35798580-36036080	IL7R	rs11954020	G>C	1.11
T1D 6q22.32	chr6:126129075-127098552	CENPW	rs1538171	G>C	1.12
		CENPW	rs9388489	A>G	1.17
		CENPW	rs9375435	G>A	1.13
T1D 6q15	chr6:90097116-90320436	BACH2	rs597325	A>G	1.18
		BACH2	rs11755527	C>G	NA*
		BACH2	rs72928038	G>A	1.20
T1D 6q27	chr6:169879562-170130554		rs924043	A>G	1.19
T1D 6q23.3	chr6:137568592-137806067	TNFAIP3	rs6920220	G>A	1.12
T1D 6q25.3	chr6:158897742-159119181	TAGAP	rs1738074	A>G	1.10
T1D 7p15.2	chr7:26618343-27162670		rs7804356	G>A	1.14
T1D 7p12.1	chr7:50833203-51066332	COBL	rs4948088	A>C	1.30
T1D 7p12.2	chr7:50327041-50624014	IKZF1	rs62447205	G>A	1.12
T1D 9p24.2	chr9:4238265-4315442	GLIS3	rs10758593	G>A	1.15
		GLIS3	rs7020673	G>C	1.14
		GLIS3	rs6476839	T>A	1.12
T1D 10p11.22	chr10:32983821-33222119	NRP1	rs722988	A>G	NA*
T1D 10p15.1	chr10:6388017-6503142	PRKCQ	rs11258747	C>A	1.13
T1D 10p15.1	chr10:5988280-6146375	RBM17 IL2RA	rs2104286	G>A	1.30
		RBM17 IL2RA	rs10795791	A>G	1.16
		RBM17 IL2RA	rs41295121	A>G	2.04
		RBM17 IL2RA	rs61839660	A>G	1.61
		RBM17 IL2RA	rs12251307	A>G	1.33
		RBM17 IL2RA	rs7090530	C>A	1.22
T1D 10q23.31	chr10:88245291-88511262	RNLS	rs10509540	G>A	1.22
		RNLS	rs12416116	A>C	1.18
T1D 11p15.5	chr11:2092701-2260001	INS	rs689	T>A	2.38
		INS	rs72853903	A>G	1.18
		INS	rs7111341	G>A	NA*
T1D 11p15.5	chr11:2026410-2051274	INS	rs7928968	A>T	1.25

T1D 11q13.1	chr11:64076473-64456055	BAD	rs694739	G>A	NA*
T1D 12q13.13	chr12:53058065-53209207	ITGB7	rs11170466	G>A	1.19
T1D 12q13.2	chr12:55957562-56404651	IKZF4 ERBB3 DGKA	rs705705	C>G	1.25
		IKZF4 ERBB3 DGKA	rs705704	G>A	1.35
		IKZF4 ERBB3 DGKA	rs11171739	A>G	1.34
		IKZF4 ERBB3 DGKA	rs2292239	C>A	1.28
T1D 12q24.13	chr12:111278572-112592683	NAA25 SH2B3	rs3184504	G>A	1.27
		NAA25 SH2B3	rs17696736	A>G	1.28
		NAA25 SH2B3	rs653178	A>G	1.30
T1D 12p13.31	chr12:9366576-9820167	CD69	rs4763879	G>A	1.09
T1D 13q32.3	chr13:99240634-99534324	GPR183	rs9585056	A>G	1.12
T1D 14q32.2	chr14:97895009-98138364		rs4900384	A>G	1.09
			rs1456988	A>C	1.12
T1D 14q24.1	chr14:68071518-68485527	RAD51B	rs911263	G>A	1.12
T1D 14q24.1	chr14:68696738-68851345	ZFP36L1	rs1465788	A>G	1.16
T1D 15q25.1	chr15:78709357-78968794	CTSH	rs3825932	G>A	1.16
		CTSH	rs34593439	A>G	1.28
		CTSH	rs12148472	G>A	1.20
T1D 15q14	chr15:38522176-38701912	RASGRP1	rs72727394	G>A	1.15
T1D 16p11.2	chr16:28283985-29014657	IL27	rs4788084	A>G	1.16
		IL27	rs9924471	G>A	1.24
		IL27	rs151234	C>G	1.19
T1D 16p13.13	chr16:10923201-11372654	CLEC16A DEXI	rs12708716	G>A	1.20
		CLEC16A DEXI	rs193778	A>G	1.14
		CLEC16A DEXI	rs12927355	A>G	1.23
T1D 16q23.1	chr16:75182342-75487132		rs8056814	G>A	1.29
			rs7202877	A>C	1.28
T1D 17q21.31	chr17:45903164-46788073		rs1052553	G>A	1.12
T1D 17q21.2	chr17:40563547-40722575		rs7221109	A>G	1.05
T1D 17q12	chr17:39226421-40084508	GSDMB ORMDL3	rs12453507	C>G	1.11
		GSDMB ORMDL3	rs2290400	G>A	1.15
T1D 18q22.2	chr18:69831437-69902924	CD226	rs763361	G>A	1.16
		CD226	rs1615504	G>A	1.13
T1D 18p11.21	chr18:12738414-12925254	PTPN2	rs1893217	A>G	1.21
		PTPN2	rs2542151	A>C	1.30
T1D 19q13.33	chr19:48592641-48763133	FUT2	rs516246	G>A	1.15
		FUT2	rs602662	G>A	1.12
T1D 19q13.32	chr19:46648121-46811943		rs425105	G>A	1.16
			rs402072	G>A	1.15
T1D 19p13.2	chr19:10280033-10517872	TYK2	rs34536443	G>C	1.49
		TYK2	rs12720356	C>A	1.22
T1D 20p13	chr20:1508984-1741341		rs2281808	A>G	1.11
T1D 21q22.3	chr21:42389067-42458550	UBASH3A	rs11203203	G>A	1.14
		UBASH3A	rs11203202	C>G	1.16
T1D 22q12.2	chr22:29670355-30273198	HORMAD2	rs5753037	G>A	1.10
		HORMAD2	rs4820830	A>G	1.14
T1D 22q12.3	chr22:37171803-37262764	RAC2 C1QTNF6	rs229533	A>C	1.11
T1D Xq28	chrX:154425779-155057576	GAB3	rs2664170	A>G	1.16

Type 1 diabetes-associated SNP variants obtained from www.immunobase.org. rs10492166, rs11171710, rs12908309, rs56994090, rs6043409, rs6691977, rs6916742, rs9272346, rs941576 were not included on the Infinium ImmunoArray-24 v2 BeadChip Kit. rs9268645 is located in HLA and was therefore excluded from the analysis.
*Excluded from GRS because odds ratio could not be found in the indicated reference.
T1D= type 1 diabetes, rsID=reference SNP cluster ID, OR=odds ratio, NA=not available.

ESM Table 2 – HLA DR-DQ genotypes and corresponding odds ratios

Patient	HLA-DRB1 locus 1	HLA-DRB1 locus 2	HLA-DQA1 locus 2	HLA-DQA1 locus 1	HLA-DQB1 locus 1	HLA-DQB1 locus 2	OR
Diab 065	0301	0301	0501	0501	0201	0201	10.4
Diab 066	0404	1601	0102	0301	0302	0502	5.5*
Diab 068	0301	0401	0301	0501	0201	0302	47.2
Diab 076	0301	09	0303	0501	02	02	5.3
Diab 077	0301	0403	0301	0501	02	0302	2.5
Diab 079	04	13	0102	0303	03	06	1.9
Diab 080	01	0301	0501	01	05	02	2.6
Diab 081	04	16	0102	0303	03	05	2*
Diab 083	01	0301	0501	0101	0501	02	2.6
Diab 084	12	13	0505	0505	0301	0301	0.7*
Diab 085	0301	0401	0301	0501	02	0302	47.2
Diab 086	0301	11	0505	0501	02	0301	0.2
Diab 087	01	16	0102	0101	05	05	1.5
Diab 088	0301	0401	0301	0501	02	0302	47.2
Diab 089	0301	0404	0301	0501	0201	0302	16.9
Diab 090	03	08	0401	0501	2	04	1.6
Diab 091	0301	0401	0301	0501	0201	0302	47.2
Diab 092	0301	0404	0301	0501	0201	0302	16.9
Diab 093	0301	0401	0301	0501	0201	0302	47.2
Diab 094	01	01	0101	0101	05	05	1
Diab 095	0101	0301	0501	0101	0501	0201	2.6
Diab 096	0301	0401	0301	0501	0201	0302	47.2
Diab 100	0301	0401	0301	0501	0201	0302	47.2
Diab 101	0401	1301	0102	0301	0302	0604	7
Diab 103	0301	0404	0301	0501	0201	0302	16.9
Diab 104	0801	1601	0102	0401	0402	0502	1.6*
Diab 105	0301	0403	0301	0501	0201	0302	2.5
Diab 106	0301	0401	0301	0501	0201	0302	47.2
Diab 107	0301	0403	0301	0501	0201	0302	2.5
Diab 108	0401	0701	0201	0301	0302	0202	8.2
Diab 109	0301	0301			0201	0201	10.4
Diab 110	0301	0401	0301	0501	0201	0302	47.2
Diab 111	0301	0401	0301	0501	0201	0302	47.2
Diab 112	0301	0301	0501	0501	0201	0201	10.4
Diab 113	0101	0301	0101	0501	0201	0501	2.6
Diab 114	0401	1101	0301	0505	0301	0302	2.2
Diab 115	0301	1501			0201	0602	0.1
Diab 116	0301	0404	0301	0501	0201	0302	16.9
Diab 125	0301	0404	0303	0501	0201	0302	16.9
Diab 126							NA
Diab 127							NA

Diab 128	0401	1301	0103	0301	0302	0603	7
Diab 129	0401	1301	0103	0301	0302	0603	7
Diab 130	0101	0301	0101	0501	0201	0501	2.6
Diab 132	0301	1301	0102	0501	0201	0604	1.3
Diab 134	0401	0401	0301		0302	0302	11.9
Diab 135	0301	1001	0105	0501	0201	0501	1*
Diab 136	0403	0403	0301		0302	0302	0.5*
Diab 137	0101	0401	0101	0301	0302	0501	6.5
Diab 138	0101	0401	0101	0303	0302	0501	6.5
Diab 139	0301	1301	0102	0501	0201	0604	1.3
Diab 140	0301	0401			0201	0302	47.2
Diab 141	0301	0401	0303	0501	0201	0302	47.2
Diab 142	0801	1301	0102	0401	0402	0604	0.7
Diab 143	1601	1301	0102	0102	0502	0603	1*
Diab 144	0101	0401	0101	0301	0302	0501	6.5
Diab 145	0401	0701	0201	0301	0201	0302	8.2
Diab 146	0301	0401	0301	0501	0201	0302	47.2
Diab 147	0701	0901	0201	0302	0201	0303	0.9*
Diab 148	0302	0404	0303	0401	0302	0402	8.4*
Diab 149	0401	0401	0301		0302	0302	11.9
Diab 150	0301	0401	0301	0501	0201	0302	47.2
Diab 151							NA
Diab 152	0101	0401	0101	0301	0302	0501	6.5
Diab 153	0301	0404	0303	0501	0201	0302	16.9
Diab 154	0301	1101	0505	0505	0201	0301	0.2
Diab 155	0301	0404	0301	0501	0201	0302	16.9
Diab 156	0404	0701	0201	0301	0201	0302	1*
Diab 157	0401	0401	0301		0302	0302	11.9
Diab 158	0401	1601	0102	0301	0302	0502	5*
Diab 159	0701	1301	0103	0201	0201	0603	0.8
Diab 160	0301	1301	0102	0501	0201	0604	1.3*
Diab 161	0301	0402	0301	0501	0201	0302	15.2
Diab 162	0301		0501	0501	0201		10.4
Diab 163	0301	0401	0301	0401	0201	0302	47.2
Diab 164	0402	1301	0102	0301		0604	2*
Diab 165	0301	0401	0301	0501	0201	0302	47.2
Diab 166	0301	0401	0301	0501	0201	0302	47.2
Diab 167	0301	1601			0201	0502	3.1
Diab 168	0101	0801	0401	0101	0501	0402	0.7

Type 1 diabetes odds ratios for HLA DR-DQ haplotypes as reported in (3).

*Estimated odds ratios (not known from literature).

OR=odds ratio, NA=not available.

ESM Figure 1

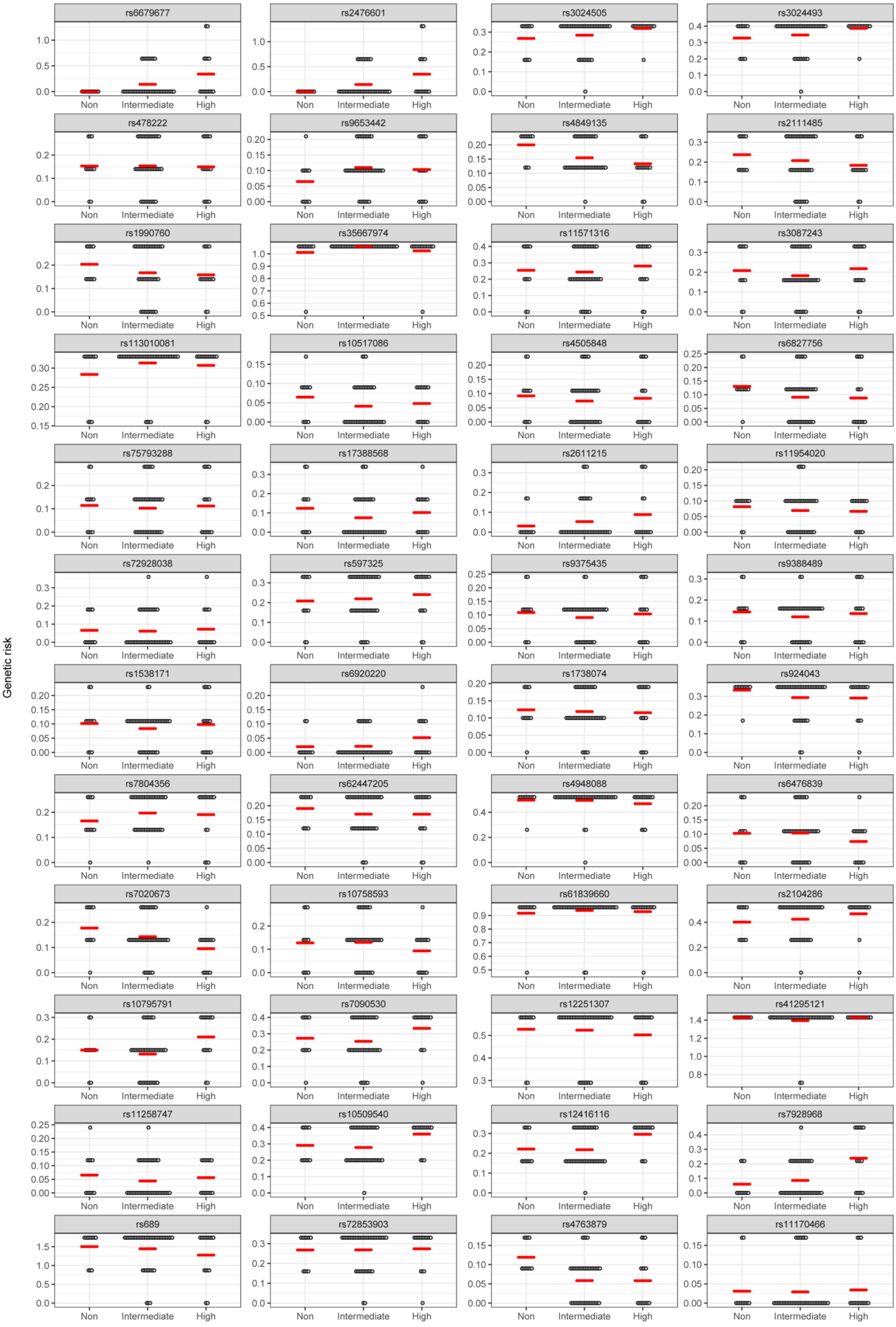
rs6679677
rs2476601
rs3024505
rs3024493
rs478222
rs9653442
rs4849135
rs2111485
rs1990760
rs35667974
rs11571316
rs3087243
rs113010081
rs10517086
rs4505848
rs6827756
rs75793288
rs17388568
rs2611215
rs11954020
rs72928038
rs597325
rs9375435
rs9388489
rs1538171
rs6920220
rs1738074
rs924043
rs7804356
rs62447205
rs4948088
rs6476839
rs7020673
rs10758593
rs61839660
rs2104286
rs10795791
rs7090530
rs12251307
rs41295121
rs11258747
rs10509540
rs12416116
rs7928968
rs689
rs72853903
rs4763879
rs11170466
Genetic risk
Non
Intermediate
High

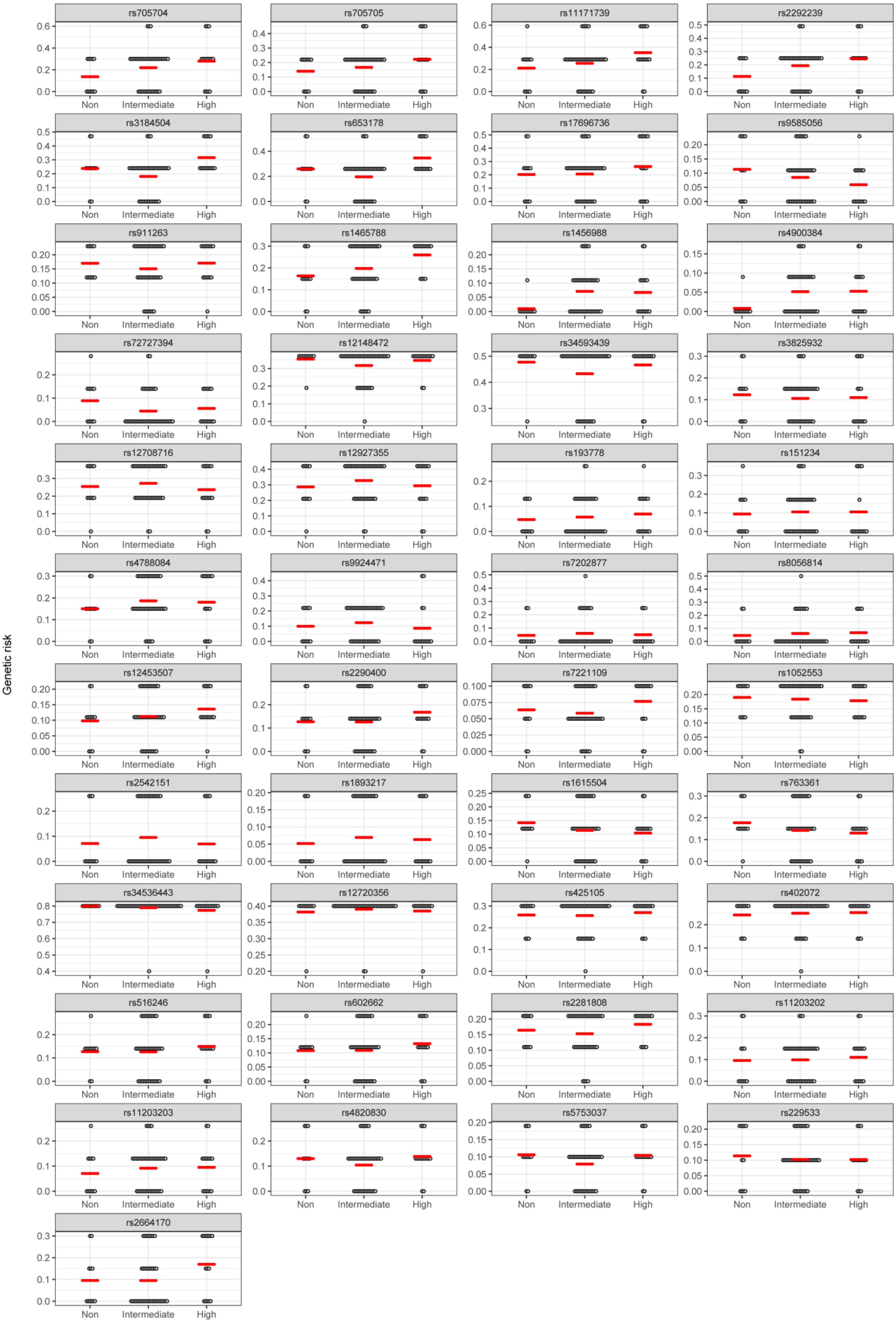

Genetic risk
rs705704
rs705705
rs11171739
rs2292239
rs3184504
rs653178
rs17696736
rs9585056
rs911263
rs1465788
rs1456988
rs4900384
rs72727394
rs12148472
rs34593439
rs3825932
rs12708716
rs12927355
rs193778
rs151234
rs4788084
rs9924471
rs7202877
rs8056814
rs12453507
rs2290400
rs7221109
rs1052553
rs2542151
rs1893217
rs1615504
rs763361
rs34536443
rs12720356
rs425105
rs402072
rs516246
rs602662
rs2281808
rs11203202
rs11203203
rs4820830
rs5753037
rs229533
rs2664170
Non
Intermediate
High

Genetic risk per SNP variant in immune-responders. Genetic risk per SNP variant per responder-group. For each SNP variant, dots indicate the genetic risk per patient: $Patient_x - SNP_i = ln(OR_{SNPi}) * copy_{SNPi}$, where $Patient_x$ refers to an individual patient, SNP_i to an individual SNP, $ln(OR_{SNPi})$ to the natural logarithm of the OR of that SNP and $copy_{SNPi}$ to the number of risk alleles the patient has for that SNP. Horizontal red bars indicate group means. Non=non-responders, Intermediate=Intermediate responders, High=high responders.

ESM Figure 2

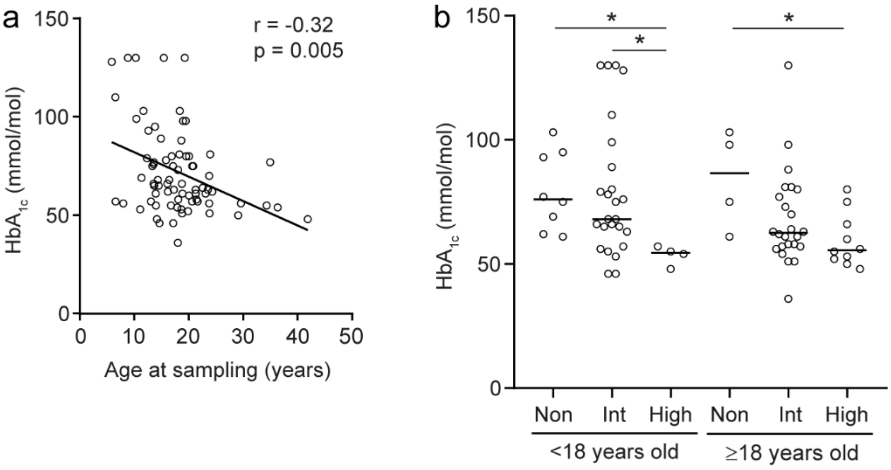

Islet autoimmunity and HbA1c are not confounded by age at blood sampling. (A) Spearman correlation between HbA1c and age at blood sampling in 75 individuals. (B) Age at blood sampling per responder-group for individuals stratified into teenagers (<18 years, n=37) and adults (≥18 years, n=38). Horizontal bars indicate group medians. Significance was tested using Dunn's multiple comparison test. Non=non-responders, Int=intermediate responders, High=high responders. *p<0.05.
